# A short generic patient experience questionnaire: *howRwe* development and validation

**DOI:** 10.1186/s12913-014-0499-z

**Published:** 2014-10-22

**Authors:** Tim Benson, Henry WW Potts

**Affiliations:** R-Outcomes Ltd, 14 Pinewood Crescent, Hermitage, Berkshire RG18 9WL UK; CHIME, University College London, 222 Euston Road, London, NW1 2DA UK

**Keywords:** Patient experience questionnaire, howRwe, Patient-reported outcome measures, Patient-reported experience measures

## Abstract

**Background:**

Patient experience is a key quality outcome for modern health services, but most existing survey methods are long and setting-specific. We identified the need for a short generic questionnaire for tracking patient experience.

**Methods:**

We describe the development and validation of the *howRwe* questionnaire. This has two items relating to clinical care (*treat you kindly*; *listen and explain*) and two items relating to the organisation of care (*see you promptly*; *well organised*) as perceived by patients. Each item has four responses (*excellent*, *good*, *fair* and *poor*). The questionnaire was trialled in 828 patients in an orthopaedic pre-operative assessment clinic (PAC).

**Results:**

The *howRwe* questionnaire is shorter (29 words) and more readable (Flesch-Kincaid grade score 2.2) than other questionnaires with broadly similar objectives. Psychometric properties in this sample are good with Cronbach’s α=0.82. Following a change to the appointments system in the clinic, *howRwe* showed improvement in promptness and organisation, but not in kindness and communication, showing that it can distinguish between the clinical and organisational aspects of patient experience.

**Conclusions:**

*howRwe* meets the criteria for a short generic patient experience questionnaire that is suitable for frequent use. In the validation study of PAC patients, it showed good psychometric properties and concurrent, construct and discriminant validity.

## Background

Patient experience is a key quality outcome for health services and can be used to improve quality, governance, public accountability and patient choice [1]. Since the pioneering work during the 1980s [2,3], the use of patient experience surveys has grown enormously, but there is little evidence of their impact on quality improvement at the local level [4].

Large-scale national surveys address the needs of policymakers for accountability and transparency [5], but there is said to be a “chasm” between the views of senior managers and clinicians at the front line [6]. Traditional methods used have been criticised for survey length, infrequent sampling frequency, slow feedback and failure to use results to improve care [7].

In England the NHS undertakes national surveys of patient experience for inpatient, outpatient, accident and emergency, maternity, mental health and general practice care sectors. Each questionnaire is around 3000 words long and mailed to several hundred patients from each provider. Response rates vary from 34% (General Practice patient survey with one reminder [8]) to 49% for the Inpatient survey (two reminders) [9]. Response rates have declined over the years from 64% in 2001 [10].

At the opposite extreme from long surveys, the Friends and Family Test (FFT) is being introduced across all NHS services [11]. This has a single global question (*how likely are you to recommend this provider to friends of family if they needed similar care or treatment?*) with six possible responses (from *extremely likely* to *extremely unlikely* plus *don’t know*) and a free text comment box. A review of the first year of operation showed that the FFT (and in particular the free text comments) could be useful for service improvement by promoting a culture of increased responsiveness if patient feedback is provided in near real time [12]. However, statistical and methodological problems mean that the FFT should not be regarded as a survey instrument, is not suitable as a comparator across organisations [13], or as a basis for incentive payments. As a single global rating, the FFT cannot show differences between aspects of patient experience and summary scores may have advantages [14].

Some providers have invested in survey systems to support locally designed questionnaires, but these require substantial local effort in design, data collection, analysis and interpretation. Unless questions are standardised, the results are of limited value for comparison, benchmarking and tracking progress. The total costs are often underestimated or unknown.

There is little consensus about what patient experience is [15]. Patient experience, satisfaction, perception, engagement, participation, preferences and outcome are distinct concepts, but only experts appreciate the distinctions. The Beryl Institute’s broad definition of patient experience as: “the sum of all interactions, shaped by an organization’s culture, that influence patient perceptions, across the continuum of care” [16] does not help much when it comes to measure it.

Short survey instruments reduce the users’ burden and various efforts have been made to create short form versions from longer survey instruments, although the difficulties are often underestimated [17].

Most patient experience questionnaires are specific to a particular setting, such as general practice, inpatient, outpatient, maternity, care home or domiciliary care, which limits their use in evaluation across different settings. On the other hand, generic instruments allow comparisons between settings along the patient pathway. However, the use of generic patient experience instruments remains rare.

We identified the need for a short generic patient experience measure to capture patients’ perception of their experience with minimal effort and to provide rapid feedback to all stakeholders in a way that is comparable, scalable and economic.

This paper describes the development and testing of this new instrument, called *howRwe*.

## Methods

### Development of questionnaire

The development of *howRwe* began in 2009. The design criteria were similar to those required for patient-reported quality of life measures [18,19], and in particular that it should be clear, brief, generic, suitable for frequent use, support multi-modal data collection, be responsive and have good psychometric properties.

1. *Clear***–** the wording should be simple and unambiguous, so that the instrument can be readily understood by vulnerable people and translated accurately into other languages.
2. *Brief* – the instrument should be short and hence quick to use by patients or their proxies, if patients are too ill to complete it themselves.
3. *Generic* – the instrument should be generic, applicable without change across all patient categories and care settings, including primary, secondary, community, emergency, domiciliary and social care.
4. *Frequent use* – the instrument should be suitable for frequent and repeated use.
5. *Multimodal* – data collection modalities should include paper, touchscreen devices such as kiosks, smartphones and tablets, web browsers and telephones including automated interactive voice response (IVR) systems.
6. *Responsive* – the instrument should be sensitive to changes and only include items under the day-to-day control of local staff and management. It should exclude aspects, such as location, transport, car-parking, payments and other regulations that cannot easily be changed.
7. *Psychometrics* – the instrument should have good psychometric properties, including validity and reliability.

In addition to these criteria, we wanted the instrument to provide scores for each dimension and a summary score. Results should be easy to understand and interpret by all stakeholders. Feedback should be provided in near real time to enable immediate remedial action by clinicians and managers.

Finally, we wanted the instrument to have a broadly similar look and feel to that of our *howRu* patient-reported outcome measure (PROM) [20].

The core premise of *howRwe* is that all patients want high quality service from staff and from the organisation as a whole. Patient experience can be classified in terms of relationships with staff and system function [21]. The EUROPEP project used the terms *clinical behaviour* and *organisation of care* in evaluating general practice care [22]. Clinical behaviour covers interactions with staff such as kindness and communication; patients are good judges of these relationships. Organisation of care covers access, waiting times, reliability and efficiency; good staff may be let down by poor systems.

The methods used to develop and test the wording included extensive desk research and literature reviews, informal focus groups with patients and staff, and pilot studies over a five-year period across a range of health and social care settings including hospitals, GP surgeries, community services and care homes. Our approach was influenced by agile software development methods [23]. Prototypes were tested and numerous improvements made. This process evolved through more than 50 distinct versions, with numerous minor changes, testing and refinements.

One of the challenges was to find short generic phrases applicable to all types of patient and setting. For example, we often refer to health and care professionals using setting-specific terms such as GP, physician, surgeon, dentist, nurse, paramedic and social worker. We avoided this issue by not referring explicitly to any staff.

Building on previous experience with *howRu*, we adopted the same pattern of using four short questions, each with four responses. We began by using the same responses as *howRu* (*none*, *a little*, *quite a lot* and *extreme*) focusing on patient concerns. However, feedback from patients and clinicians suggested that this seemed negative, so we changed the focus to excellence, with choices *excellent*, *good*, *fair* and *poor*.

**Figure 1:**
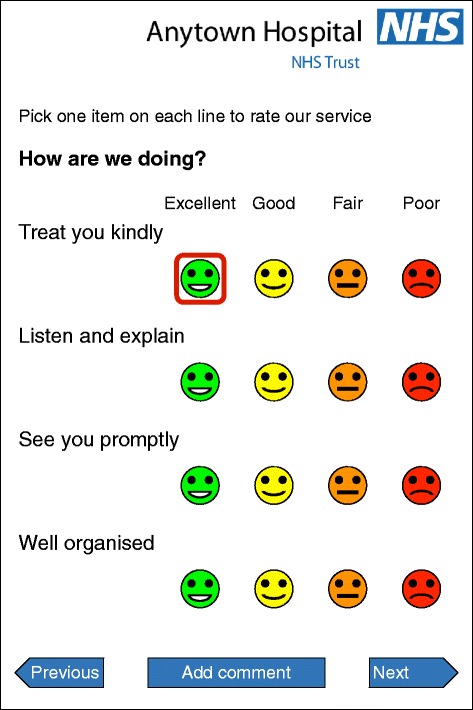
**Example of** ***howRwe*** **questionnaire configured for use on touch-screen smartphone or tablet.**

The *howRwe* questionnaire is shown in Figure 1.

The recall period applies to the present current service, because people’s memory is less reliable over longer periods [24].

The core question – *How are we doing?* – gives rise to the name of the instrument.

The descriptive system has four items. The items are short and inclusive, rather than restrictive. The items are displayed as in Figure 1 without further guidance, but we give below the intended coverage of each:

1. *Treat you kindly* (kindness) covers how you are treated as a person including compassion, empathy, emotional support, politeness, dignity, respect and privacy.
2. *Listen and explain* (communication) covers all aspects of communication with health staff including patient engagement, information, education, choice, consent, shared decision-making and empowerment.
3. *See you promptly* (promptness) covers delays, waiting, access, cancellations and responsiveness, such as the delay from referral to being seen, waiting to see a clinician, or the time taken to answer a call bell.
4. *Well organised* (organisation) covers how well managed patients perceive the unit to be, including safety, reliability, efficiency, and whether information is available when and where needed and acted on appropriately.

The strength of each item is rated using four levels:

- *Excellent*
- *Good*
- *Fair*
- *Poor*

Each level may be indicated in four mutually supporting ways to minimise cognitive effort, for face validity and avoid the need for training:

- *Written labels*: excellent*,* good*,* fair*,* poor.
- *Colour*: green, yellow, orange and red.
- *Position*: decreasing in excellence from left to right.
- *Pictographs*: based on smiley faces.

Colour, position and pictographs are optional. For example, we propose that *howRwe* can be used in voice-based systems such as interactive voice response (IVR).

The combination of four items with four levels each creates a 4 × 4 matrix with 256 (4^4^) combinations, although many of these are likely to be rare.

For analysis and reporting, each response level for each item is allocated a score on a 0–3 scale:

- *Excellent*: 3
- *Good*: 2
- *Fair*: 1
- *Poor*: 0

The summary *howRwe* score is calculated for individual respondents by adding the scores for each item, giving a scale with 13 possible values from the floor, 0 (4 × *poor*) to the ceiling, 12 (4 × *excellent*).

When reporting the results for a group comprising more than one respondent, mean scores are transformed arithmetically to a 0 to 100 scale, where 100 indicates that all respondents rated *excellent* and 0 that all rated *poor*. This allows the mean item scores to be compared with the summary *howRwe* score on a common scale.

The *howRwe* questionnaire is generic (i.e. not condition- or domain-specific) and can be used by all types of patients and citizen. If the patient is not able to complete the form personally (e.g. through dementia), a proxy such as a relative may assist or complete it on their behalf, but this should be recorded (using categories such as: *unaided*, *with help from staff*, *with help from family*, *completed by staff as proxy, completed by family as proxy*).

The *howRwe* form usually includes a comment button or text box, providing a way for respondents to add free-text comments to expand on their answers.

In this paper, we set out to test:

- *Internal consistency*: assessed by whether correlations between the four *howRwe* items are moderate to strong, with the strongest correlation between the pairs of items on clinical behaviour and organisation of care (convergent validity), and Cronbach’s α is between 0.7 and 0.9.
- *Concurrent validity*: assessed by correlation between the overall *howRwe score* and the NHS Friends and Family Test raw question.
- *Construct validity*: assessed by the measure being sensitive to system change, and system change impacting system function more than relational aspects.
- *Discriminant validity*: assessed by showing low correlation with *howRu*, a patient-reported outcome measure with a similar format.

The *howRwe* instrument is a measure of excellence. We should expect to see a ceiling effect, where service is excellent and the instrument is not able to detect further improvement. We should not expect to find a floor effect, where the service is poor and the measure cannot detect further deterioration.

### Validation

For validation, we used data from a pathfinder study in a pre-operative assessment clinic (PAC) for patients scheduled for major orthopaedic operations. This location was chosen because there were known issues and a plan was to be implemented shortly to alleviate these, providing the opportunity for before and after comparison.

Before their operation patients attend the PAC and see up to six different members of the team in the course of a morning. The purpose is to help patients prepare for their operation, discharge home and recovery. Patients are assessed for medical fitness for anaesthetic and have blood, urine and ECG tests. The surgeon explains the proposed operation. Patients also see a physiotherapist and occupational therapist and complete consent and other paperwork. All this can take several hours.

Data collection was conducted over seven months from June 2013 to January 2014 as part of routine use of the instrument. Changes to the appointment scheduling system and the physical layout of the clinic were made at the end of the third month (August).

**Figure 2:**
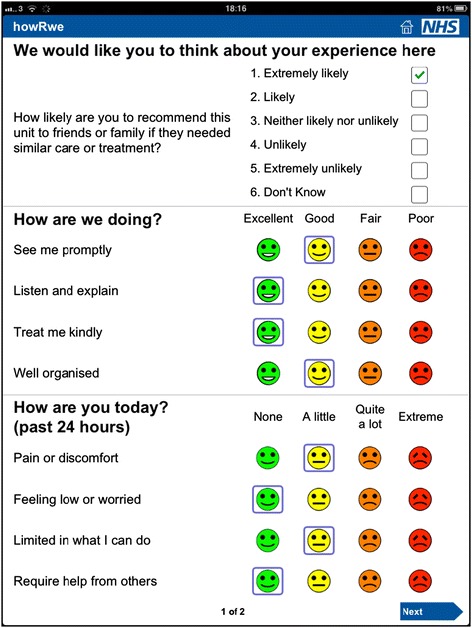
**Image of the form used on iPad in validation study.**

A member of staff asked each patient to compete the questionnaire using an iPad towards the end of their visit and recorded which surgical team and subspecialty was seen. Patients completed the NHS Friends and Family Test, *howRwe*, *howRu* and optional free-text comments. The system recorded the date and time. Data collection used a dedicated iPad linked to Optimum Health Technology’s Meridian server. An image of the form used is shown in Figure 2.

Data was exported for analysis using Excel and SPSS. We used Pearson’s correlations in the analysis.

Research ethics approval was not required because the data was collected anonymously as part of routine use of the instrument for service monitoring. No identification, demographic or medical information was collected on individual patients. All participants freely consented to complete the task.

### Readability

Questionnaire readability was measured using the Flesch-Kincaid Readability Grade (FKG) provided in Microsoft Word. The applicability of the FKG and other readability measures to questionnaire items has been questioned, but it remains a widely used tool [25]. We also offer word count as a more robust measure. As a general rule, patients should not be asked to complete questionnaires with a reading age of more than ten [26], which corresponds roughly to readability grade FKG=5.

We compared the length and readability of *howRwe* with the NHS FFT and five other patient experience questionnaires: GS-PEQ [27], EUROPEP 2006 [28], Picker PPE-15 [29], NHS Adult Inpatient Survey 2013 [30] and the GP Patient Survey 2014 [31]. For GS-PEQ and EUROPEP 2006, we used the English translations of questions and instructions as presented in original papers, which may misrepresent their performance in their original language. For the other questionnaires, we used the full text, including instructions, framing statements, questions and responses as used in surveys.

## Results

Table 1 shows the number of items, the number of words, the FKG readability grade and approximate reading age for the questionnaires assessed. *howRwe* has FKG=2.2 (reading age 7.2). The other measures have FKG in the range 6.6 to 8.8 (reading age 11.6 to 13.8), each of which is substantially greater than the criterion of FKG less than 5 (reading age 10).

**Table 1:** **Length and readability**

**Instrument**	**No of items**	**No of words**	**FKG readability grade**	**Reading age**
**howRwe**	4	29	2.2	7.2
**NHS Friends & Family Test**	1	44	6.6	11.6
GS-PEQ*	10	150	8.8	13.8
EUROPEP 2006*	23	214	8.1	13.1
**PPE-15**	15	467	7.1	12.1
**NHS adult inpatient Survey 2013**	76	3,353	7.3	12.3
**GP patient survey 2014**	62	2,922	6.8	11.8

*Note the word count and readability for GS-PEQ and EUROPEP 2006 are based on the translations of questions and instructions as presented in the original papers. For other surveys, we used the text from actual questionnaires, including instructions.

In total, 828 respondents completed all parts of the *howRwe* questionnaire. Five respondents (0.6%) answered “Don’t know” to the FFT, so their FFT scores were coded as missing. We do not have a record of how many patients were asked but declined to participate. The numbers for each orthopaedic sub-specialty are shown in Table 2.

**Table 2:** **Responses by sub-specialty**

**Sub-specialty**	**n**	**%**
**Hip and knee replacement**	354	43%
**Foot and ankle**	137	17%
**Spinal**	140	17%
**Sarcoma**	72	9%
**Shoulders**	45	5%
**Other**	80	10%
**Total**	**828**	**100%**

The distribution of responses for each item is shown in Table 3. 68% of all item responses were *excellent*, 26% *good*, 5% *fair* and 1% *poor*. As expected we found a ceiling effect with a large proportion of responses being *excellent*. The differences between items help identify aspects that need improvement.

**Table 3:** **Distribution of responses for each item (%)**

**Item**	**Excellent**	**Good**	**Fair**	**Poor**
**Treat me kindly**	671 (81.0%)	148 (17.9%)	7 (0.8%)	2 (0.2%)
**Listen and explain**	609 (73.6%)	202 (24.4%)	16 (1.9%)	1 (0.1%)
**See me promptly**	447 (54.0%)	273 (33.0%)	90 (10.9%)	18 (2.2%)
**Well organised**	530 (64.0%)	233 (28.1%)	51 (6.2%)	14 (1.7%)

The *howRwe score* is the aggregate of the four *howRwe* items. Table 4 shows the distribution of responses. 404 respondents (48.8%) rated the service as *excellent* in all respects (ceiling score), while only one (0.1%) rated it as *poor* in all respects (floor score).

**Table 4:** **Distribution of** ***howRwe*** **aggregate scores**

**howRwe score**	**n**	**%**
**12**	404	48.8%
**11**	107	12.9%
**10**	77	9.3%
**9**	56	6.8%
**8**	123	14.9%
**7**	25	3.0%
**6**	21	2.5%
**5**	8	1.0%
**4**	5	0.6%
**3**	1	0.1%
**2**	0	0.0%
**1**	0	0.0%
**0**	1	0.1%
**Total**	**828**	**100.0%**

Table 5 shows the mean patient score (raw data), the item score on a 0–100 scale, 95% confidence limits and standard deviation for each item and the aggregate *howRwe* score. *Treat me kindly* has the highest item score (93.2) and *See me promptly* the lowest (79.6).

**Table 5:** **Mean scores for each item and** ***howRwe score***

**Item**	**Mean score**	**Mean score**	**95% confidence**	**St dev**
	**(raw data)**	**(0–100 scale)**	**limits**	
**Treat me kindly**	2.80	93.2	92.2 – 94.2	14.7
**Listen and explain**	2.72	90.5	89.3 – 91.6	16.7
**See me promptly**	2.39	79.6	77.9 – 81.3	25.5
**Well organised**	2.54	84.8	83.3 – 88.1	22.9
**howRwe score**	10.44	87.0	85.9 – 88.1	16.4

The inter-item correlation matrix is shown in Table 6. The correlation between the clinical care items, *Treat me kindly* and *Listen and explain* is high (*r=* 0.71), as is the correlation between the two organisation of care items, *See me promptly* and *Well organised* (*r=* 0.70). The correlations between the other items are in the range *r=* 0.39 to *r=* 0.56.

**Table 6:** **Intra-item correlation matrix (95% confidence intervals)**

	**Listen and explain**	**See me promptly**	**Well organised**
**Treat me kindly**	0.71 (0.67, 0.74)	0.39 (0.33, 0.44)	0.51 (0.46, 0.56)
**Listen and explain**		0.47 (0.42, 0.52)	0.56 (0.51, 0.60)
**See me promptly**			0.70 (0.66, 0.73)

A factor analysis of the four items found a single factor explaining 67% of the variance, demonstrating unidimensionality. The four eigenvalues were 2.67, 0.76, 0.30 and 0.28. The internal consistency reliability was satisfactory (Cronbach’s α=0.82; 95% CI: 0.79, 0.83).

The correlation between each item and the sum of the other three items is shown in Table 7. These all lie in the range *r=* 0.60 to *r=* 0.74. The correlation of each item to the FFT question responses is also shown. These correlations are negative due to the way that the FFT is marked (good is low). These all lie in the range *r=* −0.36 to *r=* −0.48. The correlation between the aggregate *howRwe score* and the FFT question is *r=* −0.53 (−0.58, −0.48).

**Table 7:** **Correlations between each** ***howRwe*** **items and the sum of the other three items, the Friends and Family Test (FFT) question and the** ***howRu*** **summary score**

**howRwe item**	**Sum of the other three** ***howRwe*** **items**	**Item to FFT question**	**Item to** ***howRu*** **summary score**
	**(** ***r*** **)**	**(** ***r*** **)**	**(** ***r*** **)**
**Treat me kindly**	0.60 (0.56, 0.64)	−0.36 (−0.42, −0.30)	0.08 (0.01, 0.15)
**Listen and explain**	0.66 (0.62, 0.70)	−0.42 (−0.47, −0.36)	0.08 (0.01, 0.15)
**See me promptly**	0.64 (0.60, 0.68)	−0.44 (−0.49, −0.38)	−0.06 (−0.13, 0.01)
**Well organised**	0.74 (0.71, 0.77)	−0.48 (−0.53, −0.43)	0.02 (−0.05, 0.09)
***howRwe*** **summary score**	-	−0.53 (−0.58, −0.48)	0.02 (−0.05, 0.09)

The *howRwe* summary score and the *howRu* summary score (a measure of patient health status) show no significant correlation (*r*=0.02; 95% CI: −0.04, 0.09). Table 7 also shows the correlations between the individual *howRwe* items and the *howRu* summary score, which are also minimal.

**Figure 3:**
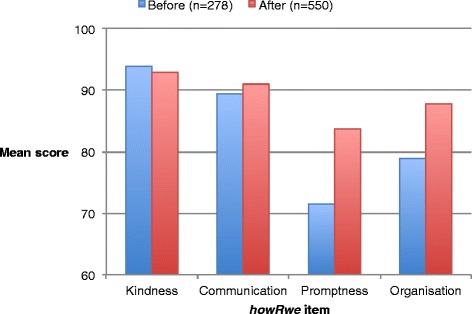
***howRwe*** **item scores before and after system change.**

Table 8 and Figure 3 show the *howRwe* item scores (on 0–100 scale) before and after changes to the appointments system. There is no significant change to the clinical care items (*Treat me kindly* and *Listen and explain*), but significant improvements in the organisation of care items (*See me promptly* and *Well organised*).

**Table 8:** ***howRwe*** **item scores before and after changes to appointments system**

***howRwe*** **Item**	**Before change**	**After change**	**Mann–Whitney test**
	**(95% confidence limits)**	**(95% confidence limits)**	
**Responses (n)**	278	550	
**Treat me kindly**	93.9 (92.2 – 95.6)	92.9 (91.7 – 94.1)	*z*=0.9, *p*=0.4
**Listen and explain**	89.4 (87.5 – 91.4)	91.0 (89.6 – 92.4)	*z*=1.0, *p*=0.3
**See me promptly**	71.5 (68.5 – 74.5)	83.7 (81.6 – 85.8)	*z*=6.1, *p*<0.0001
**Well organised**	78.9 (76.2 – 81.6)	87.8 (85.9 – 89.7)	*z*=4.7, *p*<0.0001

## Discussion

*howRwe* is the first short generic patient experience measure we know of that has been designed for use across all health and social care sectors. Its practicality at scale and in social care was demonstrated in a survey in 360 care homes in the UK, Australia and New Zealand, completed by over 10,000 residents [32]. When we began this work, we were not aware of any other short generic patient experience questionnaire, but we have since become aware of the GS-PEQ (Generic Short Patient Experience Questionnaire), which has been developed in Norway as a generic short form of the Norwegian national sector-specific surveys [27].

This is the first published report of the development of *howRwe*. We have shown that *howRwe* is shorter than other measures, has good readability statistics and internal validity.

A Cronbach’s α of 0.82 in this sample suggests that it is appropriate to use the overall *howRwe score*, as well as individual item scores [33]. Construct validity and sensitivity were shown by how scores responded to a change in the appointments system. The instrument shows minimal correlation with the *howRu* measure of quality of life, despite the shared layout.

### Development

The usual method of developing new measures is to set out a development protocol for a funded piece of work using a methodology that allows people without specific domain knowledge to develop instruments within a specified time scale.

The development of short patient experience questionnaires often start with a long measure or long sets of statements, which are refined to create a short form with far fewer items. This is how GS-PEQ and PPE-15 were developed. A different short form approach, which does not result in a new questionnaire, is to derive a scale from secondary analysis of a long form data set. The Oxford Patient Involvement and Experience scale (OxPIE) was derived in this way from the NHS Inpatient Survey 2011 [34].

Our approach was different, but allowed a new type of short generic measure to evolve.

### Study limitations

Without a gold standard for patient experience instruments and with limited consensus about most important dimensions of patient experience, it is difficult to test content validity.

The data was collected as part of routine anonymous use, rather than as a special validation study, so we do not have additional demographic or clinical data that could be used for construct validation.

The patients in this sample were undergoing a distinct episode of care. In other settings, care is extended over time and multiple healthcare staff, which may make responses from the questions asked in *howRwe* more difficult to interpret. *howRwe* has also been used successfully with hospital inpatients, general practice and community service patients and care home residents.

Although *howRwe* was designed to be applicable and comparable across multiple care sectors, this study used a relatively homogeneous respondent sample of orthopaedic patients attending a pre-operative assessment clinic, with more than 40% due to have hip or knee replacements. We recognise the long-standing debates about the relative value of generic and specific measures (*e.g.* [35,36]) and it is important to test *howRwe* in further groups.

The data was collected using a questionnaire on an iPad, which included the FFT, *howRwe* and *howRu* questions on a single screen (Figure 2). The wording of each item is identical to that shown in Figure 1, but the order is different. The reason for changing the order is to put the items about clinical care and organisation of care together. We do not consider that this change in order impacts any of the conclusions drawn.

We were not able to measure response rates in the study. Individual patients were asked to complete the questionnaire by a member of staff, who tended not to do this when very busy. We do not know how many patients declined the offer to take part, or who only completed part of the survey, because data was only submitted to the database when the questionnaire was complete. A comparable paper-based study using *howRu* obtained high completion rates [37].

In the FFT, the mode of administration and patient demographics has an impact on both response rates and scores [12]. This study did not provide any way of testing for these effects in *howRwe*.

It would be valuable to investigate the instrument’s test-retest reliability and to investigate further discriminant validity against, for example, measures of personality.

### Implications for practice

The time and effort involved in monitoring patient experience is a source of concern. Response rates for long questionnaires are falling. Patients complain of survey fatigue and the surveys are expensive to administer. For example the Guidance Manual for the 2013 Inpatient Survey runs to 34,600 words [38]. Response rates for NHS national surveys are all below 50%, in spite of up to two reminders.

The local impact of large national surveys has been less than might have been hoped. Feedback needs to be quicker, ward-specific, include patient comments and offer staff an opportunity to discuss it [39]. Existing patient experience instruments are also specific to the mode of care, making it difficult to compare across modes of treatment.

The correlation between *howRwe* and the FFT was *r=* −0.53, indicating that they are not measuring quite the same things. The FFT is a global rating of recommendation, which is related to morale, optimism and loyalty, while *howRwe* measures patient perceptions of different aspects of service. Overall summary scores, such as the *howRwe score*, may perform better than global ratings (such as the FFT) as a way of summarising patients’ experiences [14].

## Conclusions

*howRwe* is a short generic patient experience measure. The questionnaire is shorter (29 words) and more readable (Flesch-Kincaid grade score 2.2) than other widely used instruments. It minimises respondent burden, allows rapid feedback and comparisons to be made between different care settings either within an organisation or across the patient pathway. Psychometric properties are good.

*howRwe* is being used in the UK in secondary, primary, community and social care providers. It adds value by distinguishing between the aspects of patient experience that relate to clinical care (kindness and communication) and organisation of care (promptness and organisation).

We hope that future work will investigate further psychometric properties of the instrument and test its role in promoting change and improvement of quality.

## Abbreviations

FFT: Friends and Family Test
GP: General Practice/Practitioner
IVR: Interactive voice response
NICE: National Institute for Health and Care Excellence
NHS: National Health Service
PREM: Patient-reported experience measure
PROM: Patient-reported outcome measure
PAC: Pre-operative assessment clinic
